# In Vivo Degradation Behaviour and Osteoregenerative Capacity of 3D-Printed Magnesium Phosphate and Calcium Magnesium Phosphate Cement Scaffolds

**DOI:** 10.3390/ma18225067

**Published:** 2025-11-07

**Authors:** Sophia Hiepe, Elke Vorndran, Franziska Feichtner, Anja-Christina Waselau, Andrea Meyer-Lindenberg

**Affiliations:** 1 Clinic for Small Animal Surgery and Reproduction, Ludwig Maximilians University Munich, 80539 Munich, Germanyameylin@lmu.de (A.M.-L.); 2 Department for Functional Materials in Medicine and Dentistry, University of Wuerzburg, 97070 Wuerzburg, Germany

**Keywords:** degradable bone substitutes, 3D powder printing, scaffold, osteoregeneration, bone cements, struvite, farringtonite, stanfieldite

## Abstract

Developing bone substitutes that are mechanically strong, highly biocompatible and capable of controlled degradation is crucial for successful bone regeneration. Magnesium phosphate cements (MPCs) and calcium magnesium phosphate cements (CMPCs) offer higher strength and solubility than established calcium phosphate cements (CPCs). This study aimed to evaluate the in vivo degradation, osteoregeneration and biocompatibility of 3D powder-printed Mg3d (Mg_3_(PO_4_)_2_) and Mg275d (Ca_0.25_Mg_2.75_(PO_4_)_2_) scaffolds with alkaline post-treatment, using structurally identical TCP (Ca_3_(PO_4_)_2_) scaffolds as the control. The scaffolds were implanted into the lateral femoral condyle of adult female Zika rabbits and analysed up to 6, 12 and 24 weeks using radiography, microCT, histology, EDX and SEM. All materials demonstrated good biocompatibility. Mg3d and Mg275d scaffolds degraded significantly faster than the TCP scaffolds, with nearly complete degradation after 12 weeks. A cell-rich reconstruction zone formed during degradation, which was subsequently replaced by new bone. The degradation rate of the scaffolds corresponded closely to bone regeneration. Notably, the Mg3d and Mg275d scaffolds supported the faster formation of mature lamellar bone compared to the TCP scaffolds. These results indicate that magnesium phosphate (MgP)-based scaffolds represent a promising alternative to conventional calcium phosphate (CP)-based bone substitutes, given their rapid and almost complete degradation and their effective support of bone regeneration.

## 1. Introduction

Bone defects that exceed the intrinsic healing capacity of the body often require grafting. Autografts and allografts are still considered the clinical gold standard, yet their limited supply, donor site morbidity, and risk of immune reactions have prompted the search for synthetic alternatives [1,2,3]. Synthetic bone substitutes offer unlimited availability, consistent quality, and the possibility of precise adaptation to individual defect geometries [4,5]. Despite extensive research efforts, no material currently fulfills all the requirements of an ideal bone substitute. Such a material should combine biocompatibility, osteoconductivity, and controlled degradability to support bone ingrowth and to be gradually replaced by newly formed, mechanically competent tissue [5,6,7].

Among synthetic materials, calcium phosphate cements (CPCs) are well established due to their chemical similarity to the mineral phase of natural bone, which accounts for their excellent biocompatibility and osteoconductivity [6,7]. Despite these advantages, CPCs are brittle and exhibit low mechanical strength [8]. In addition, their slow resorption often leads to the persistence of residual material for extended periods, which can impede complete remodeling and increase the risk of inflammatory reactions and fractures [9,10].

To overcome these limitations, magnesium phosphate cements (MPCs) were developed as a new class of bioresorbable bone substitutes [11,12]. These materials combine improved solubility with higher mechanical stability compared to pure CPCs. Moreover, the release of magnesium ions (Mg^2+^) has been shown to enhance osteoblast proliferation, inhibit osteoclast activity and promote angiogenesis, resulting in accelerated degradation and improved osseointegration [13,14,15,16]. However, purely magnesium phosphate (MgP)-based systems may degrade too rapidly, causing temporary loss of mechanical integrity before sufficient bone formation occurs [17].

To combine these positive effects of both materials, calcium–magnesium phosphate cements (CMPCs) were introduced, combining the favorable biological activity of MPCs with the structural stability of CPCs. These materials show adjustable degradation rates, high mechanical strength, and excellent biocompatibility, making them promising candidates for resorbable bone substitutes [18,19].

Recent advances in additive manufacturing have further expanded the potential of CMPCs and MPCs. The three-dimensional (3D) powder printing process allows the cost-efficient production of patient-specific scaffolds with tailored porosity and architecture [20,21]. Their inherent microporosity (typically > 30 vol. %) promotes nutrient diffusion, cell adhesion, and vascularization [22,23]. Optimal osteoconduction occurs at pore diameters of 100–500 µm, whereas higher porosity reduces compressive strength. Therefore, bone scaffolds should ideally exhibit compressive strengths between 2 and 12 MPa, comparable to cancellous bone [14,24]. Post-processing techniques are essential for tuning the mechanical and degradation behavior of 3D-printed CMPCs and MPCs. Alkaline post-treatment with diammonium hydrogen phosphate (DAHP) promotes the formation of struvite, markedly increasing compressive strength from approximately 1.3–2.8 MPa to about 10 MPa, as demonstrated in studies by Vorndran et al. [5,25]. At the same time, the higher solubility of magnesium-substituted phases allows for better synchronization between scaffold degradation and bone regeneration compared with pure calcium phosphate materials as also confirmed in vitro [18,26].

Kowalewicz et al. [27] confirmed that the degradation rate and new bone formation depend heavily on the type of post-treatment. While CMPCs treated with alkaline post-treatment showed faster degradation, materials treated with acidic post-treatment showed more uniform osseointegration.

Kanter et al. [28] investigated the degradation mechanisms and bone regeneration capacity of struvite-forming MPC pastes in a sheep model, which showed complete degradation and trabecular bone replacement within 10 months. Kaiser et al. [17] expanded on these findings by optimizing the degradation rate of MPCs by adjusting the powder-to-liquid ratio. In a partially loaded sheep model, partial degradation of the cements and simultaneous new bone formation were demonstrated after four months.

Hemmerlein et al. [29] compared 3D-printed MPC and CMPC wedges in a partially loaded rabbit tibia model and found faster degradation for alkaline-treated MPCs than for CMPCs, corresponding to differences in their post-treatment products.

The findings of these studies highlight that the interplay between material chemistry (Ca:Mg ratio), post-treatment conditions, and pore architecture is decisive for tailoring degradation kinetics, ion exchange, and mechanical performance to achieve balanced bone regeneration. Although individual MPC and CMPC scaffolds have been widely investigated regarding their mechanical strength, porosity, degradation, and osteointegration, systematic comparisons of alternative MgP-rich formulations, such as Mg275d and Mg3d, remain to be conducted. In particular, there is a need for qualitative and quantitative characterization of their degradation behavior and the associated osteoregeneration in vivo. This study addresses this gap by investigating 3D powder-printed scaffolds that underwent alkaline post-treatment with defined pore architecture in an unloaded rabbit femoral condyle defect model. The investigated scaffold materials included Mg3d, composed of magnesium phosphate (Mg_3_(PO_4_)_2_), and Mg275d, a calcium-substituted magnesium phosphate (Ca_0_·_25_Mg_2_·_75_(PO_4_)_2_). Tricalcium phosphate (TCP) was used as a clinically established calcium phosphate reference material and served as the control in this study.

## 2. Materials and Methods

### 2.1. Production and Characterization of the Scaffold

The experimental methods used in this study are based on the protocols described by Kowalewicz et al. [19] and adapted to the specific requirements of the materials used.

#### 2.1.1. Production of the Scaffolds

Production of the cements was based on the protocols described in the preliminary studies [18,26]. Cements with the chemical composition Ca_x_Mg_3−x_(PO_4_)_2_ with x = 0 (Mg3d), x = 0.25 (Mg275d) und x = 3 (TCP) were produced from calcium hydrogen phosphate (CaHPO_4_, J.T. Baker, Phillipsburg, NJ, USA), calcium carbonate (CaCO_3_, Merck, Darmstadt, Germany), magnesium hydrogen phosphate (MgHPO_4_-3H_2_O, Alfa Aesar, Kandel, Germany) and magnesium hydroxide (Mg(OH)_2_, VWR International GmbH, Darmstadt, Germany) in molar ratios corresponding to (Table 1).

**Figure 1:**
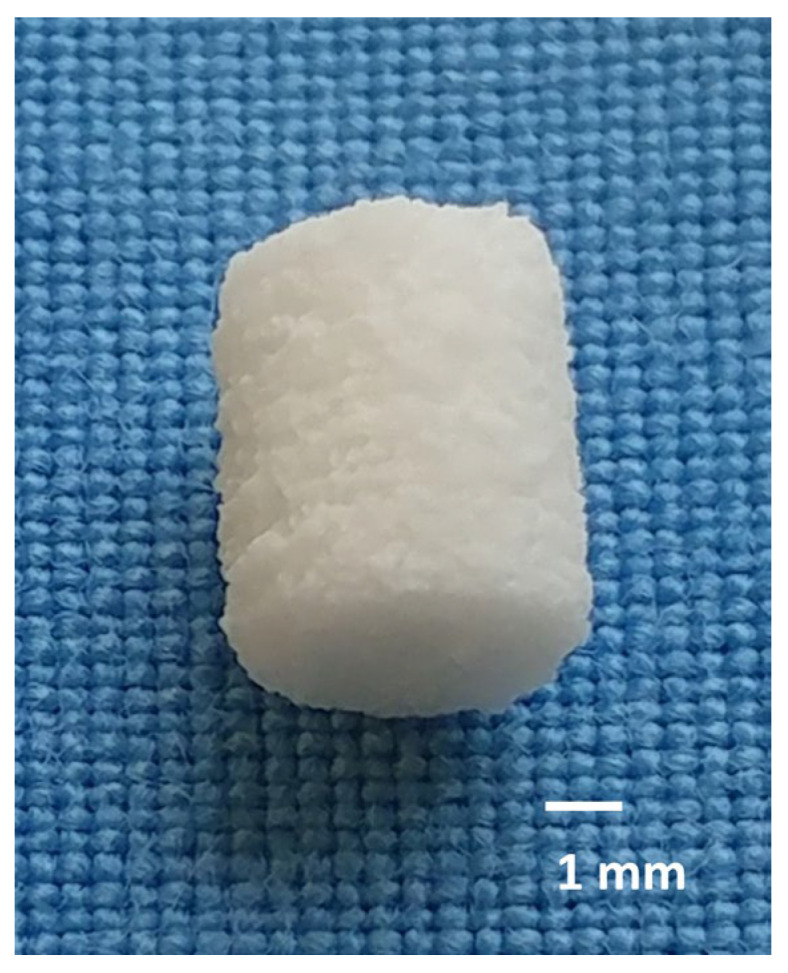
The scaffold prior to implantation (h = 5.1 mm, Ø = 4.2 mm).

The raw materials were sintered at 1100–1400 °C, ground and then sieved to a particle size of <355 µm. The cylindrical scaffolds (h = 5.1 mm, Ø = 4.2 mm) were 3D-printed using a 3D powder printer (Z-Corporation, Burlington, MA, USA) mixed with 4 wt% hydroxypropyl methylcellulose. After printing, the scaffolds were dedusted, cellulose was burned out at 500 °C for 2 h and sintered. In the case of Ca_0.25_Mg_2.75_(PO_4_)_2_ sintering was carried out at 1100 °C, while Mg_3_(PO_4_)_2_ was sintered at 1200 °C. Ca_3_(PO_4_)_2_ was sintered for 4 h at 1350 °C and in a further sintering process to convert α- to ß-tricalcium phosphate for 4 h at 1000 °C. After the sintering process, the Mg3d and Mg275d scaffolds underwent alkaline post-treatment. They were stored for 24 h in 3.5 M (NH_4_)_2_HPO_4_ solution. Sintered TCP scaffolds were used without post-treatment and served as clinically established reference material. Scaffolds (Figure 1) were washed, dried and γ-sterilized (>25 kGy, BBF Sterilisationsservice GmbH, Kernen, Germany) prior to implantation.

#### 2.1.2. Analysis of Chemical Composition

The chemical composition of the scaffolds was investigated by X-ray diffractometry (D8 Advance, Bruker Corporations, Karlsruhe, Germany) in combination with Rietveld analysis.

The phases were identified using DIFFRAC.EVA V.5.1.0.5. software (Bruker Corporations, Billerica, MA, USA). The quantitative phase analysis was performed based on the diffractograms using TOPAS V6 software (Bruker Corporations, Billerica, MA, USA).

#### 2.1.3. Compressive Strength of the Scaffolds and Young’s Modulus

The compressive strength test was carried out in accordance with the specifications of ISO standard 13175-3:2012 [30]. The compressive strength was determined for each material using ten samples that had been stored for 24 h at 37 °C in phosphate-buffered saline (PBS). The measurements were performed using a testing machine (Z010, Zwick GmbH, Ulm, Germany) equipped with a 10 kN load cell. A small preload of 1 N was applied to eliminate gaps between specimen and the testing platens. The test speed was 1 mm/min. Young’s modulus was calculated from the stress–strain slope.

#### 2.1.4. Porosity of the Scaffolds

The porosity of the materials was determined by three measurements per group. The Pascal 140/440 porosimeter (Thermo Fisher Scientific Inc., Waltham, MA, USA) was used for this purpose. The measurements were taken within a pressure range of 0.01 kPa to 400 MPa. The data was evaluated using SOLID software (SOLver of Intrusion Data Ver. 1.6.5, Thermo Fisher Scientific Inc., Waltham, MA, USA).

#### 2.1.5. Energy Dispersive X-Ray (EDX) and Scanning Electron Microscopy (SEM) Prior to Implantation

The scaffolds were fixed in 4% buffered formalin solution, then dehydrated in an ascending alcohol series (Roth, Karlsruhe, Germany) and degreased with xylene (Roth, Karlsruhe, Germany). They were then embedded in a methyl methacrylate-based plastic system (Technovit^®^ 9100, Heraeus Kulzer, Wehrheim, Germany). A central thin section (4 µm) was prepared using a rotary microtome (RM2255 Leica, Wetzlar, Germany) and mounted on glass slides coated with ponal-poly-L-lysine (Glaswarenfabrik Karl Hecht, Sondheim, Germany). The samples were smoothed, dried and fixed in a slide press at 37 °C.

For SEM analysis, the samples were coated with platinum layer and examined using a field emission electron microscope (Crossbeam CB 340, Zeiss, Oberkochen, Germany). The chemical phase distribution was analysed using EDX (INCA Energy 350 AzTec Advanced System, Oxford Instruments, Abingdon, UK) at an acceleration voltage of 5–10 keV. The pore structure and the chemical elements magnesium (Mg), calcium (Ca) and phosphate (P) were qualitatively evaluated.

### 2.2. In Vivo Study

This study was approved by the government of Upper Bavaria in accordance with paragraph 8 of the Animal Welfare Act (approval number Az. ROB 55.2-2532.Vet_02-19-64). A total of 36 adult female Zimmermann rabbits (ZIKA rabbits, Asamhof, Kissing, Germany) aged 6.1 months on average and weighing 4.3 kg on average were included in the study. The rabbits were housed individually in climate-controlled facilities (15–21 °C, 45–65% humidity, 12:12 h light–dark cycle) in cages equipped with a drinking bottle, feed bowl, hay rack, a shelter, and enrichment. A 14-day acclimatization period was observed, and animals received controlled group exercise (daily 10 min, twice weekly 60 min). The rabbits were divided into three observation groups (6, 12, and 24 weeks, n = 12 animals/group). Each rabbit received two scaffolds, one in each lateral femoral condyle. Three scaffold types (Mg3d, Mg275d, TCP) were investigated, with each type implanted 24 times in total (n = 8 per material and time group).

#### Surgery

**Figure 2:**
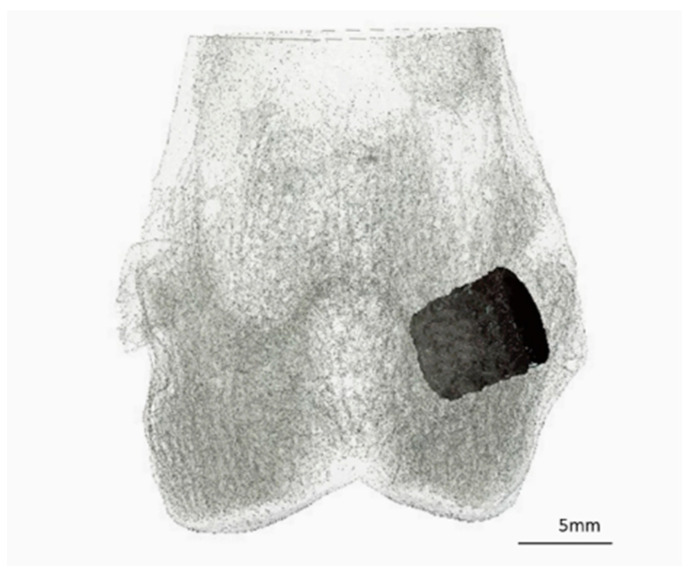
Three-dimensional (3D) reconstruction of the distal rabbit femur with implanted scaffold in the lateral condyle in frontal view.

One hour before surgery, the animals were given an antibiotic (enrofloxacin 10 mg/kg, Enrobactin^®^, CP-Pharma GmbH, Burgdorf, Germany) and analgesic treatment (meloxicam 0.3 mg/kg, Rheumocam^®^, Albrecht GmbH, Aulendorf, Germany) orally. The operation was performed under general anaesthesia, which was induced with medetomidine (0.25 mg/kg i.m., Dorbene vet^®^, Zoetis Deutschland GmbH, Berlin, Germany) and ketamine (15 mg/kg i.m., Anesketin^®^, Albrecht GmbH, Aulendorf, Germany). The animals were then endotracheally intubated, the surgical site shaved and prepared aseptically. Anaesthesia was maintained with isoflurane (1.5–2 vol%, with simultaneous oxygen supply of 1 L/min). Throughout the operation, the rabbits received fentanyl (10 µg/kg/h i.v., Fentadon^®^, Albrecht GmbH, Aulendorf, Germany) via a syringe pump (Perfusor^®^ compact, B. Braun AG, Melsungen, Germany) for analgesia. The surgical procedures were performed as described by Kowalewicz et al. [19,27]. The scaffold was implanted precisely into a drill hole defect in the spongy area of the lateral femoral condyle (Figure 2). Immediately after surgery, in vivo microcomputed tomography (µCT) was performed on both hind limbs and X-rays were taken in two projections. Postoperatively, the animals received buprenorphine (20 µg/kg i.v., Bupresol^®^, CP-Pharma GmbH, Burgdorf, Germany). The antibiotic enrofloxacin (10 mg/kg, Enrobactin^®^, p.o., CP-Pharma GmbH, Burgdorf, Germany) and the analgesic meloxicam (0.3 mg/kg p.o., Rheumocam^®^, Albrecht GmbH, Aulendorf, Germany) were administered to the animals once daily for five days after the operation. In the first two weeks after surgery, the animals received close daily clinical examination, encompassing evaluations of overall condition, appetite and hydration, gastrointestinal and urinary output, body weight fluctuations, and any evidence of pain, lameness, or wound-healing disorders. In the following weeks, the animals were clinically examined every other day. After the respective trial period (6, 12, or 24 weeks), the animals were euthanized with pentobarbital (200–230 mg/kg, Narkodorm^®^, CP-Pharma GmbH, Burgdorf, Germany) in accordance with animal welfare guidelines after sedation with propofol (5 mg/kg, Narcofol^®^, CP-Pharma GmbH, Burgdorf, Germany). The femora were obtained, and any attached soft tissue was carefully removed.

### 2.3. X-Ray Examinations

Immediately after surgery and at fixed intervals (every 2 weeks until week 12, then every 4 weeks), the knee with the implanted scaffolds were X-rayed in two projections (cranio-caudal (cr/cd) and medio-lateral (m/l)) using an X-ray machine (Multix Select DR, Siemens GmbH, Erlangen, Germany). For this purpose, the animals were sedated with medetomidine (0.25 mg/kg i.m., Dorbene vet^®^, Zoetis Deutschland GmbH, Berlin, Germany) and ketamine (115 mg/kg–0.5 kg i.m., Anesketin^®^, Albrecht GmbH, Aulendorf, Germany). The settings 54.9 kV and 4.5 mAs were used for the images. The images were evaluated using DicomPACS vet software (Ver. 8.3.20, Oehm und Rehbein GmbH, Rostock, Germany). Both projections were evaluated independently by two examiners for visibility of the scaffold.

### 2.4. In Vivo µCT Examinations

After the X-ray examination the still sedated animals were in a supine position with their hind limbs extended and supplied with oxygen (2 L/min) via a laryngeal mask (v-gel^®^ rabbit, Docsinnovet Ltd., London, UK). The µCT scans from the knee joint space to the proximal femoral condyles were performed using the XtremeCT (Scanco Medical, Zurich, Switzerland). The settings used were 68 kV voltage, 1000/180° projections, 200 ms integration time and voxel size 30.3 µm. All µCT scans were displayed and calculated using the µCT Evaluation Program V6.6:403 software (Scanco Medical, Zurich, Switzerland). The original scans were rotated along the longitudinal axis of the scaffolds, thereby displaying the scaffold cross-section.

#### 2.4.1. Quantitative Evaluation of In Vivo μCT Scans

In order to distinguish the scaffold from the surrounding bone tissue, a separate threshold (Th) was determined for each material based on the gray values of six implanted scaffolds immediately after surgery, as described in the literature [19]. The thresholds were 103 for Mg3d, 121 for Mg275d, 219 for TCP and 142 for cancellous bone at the same location.

**Figure 3:**
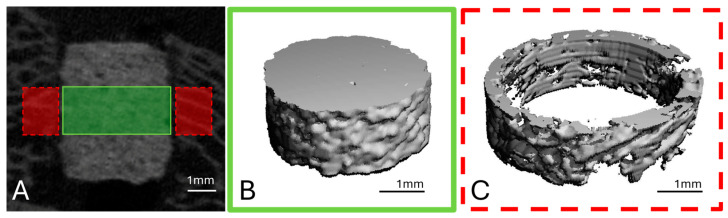
(**A**) Microcomputed tomography (µCT) scan of the implanted scaffold on the day of surgery. The measurement area in the middle third of the scaffold (ROI: height = 1.82 mm; ø = 4.24 mm) for calculating the SV is shown in green. The red dotted line indicates the measurement area of the scaffold environment (ROI: height = 1.82 mm; inner diameter = 4.36 mm; outer diameter = 5.45 mm), which was used to calculate BV and Tb.N. (**B**) 3D reconstruction of the middle scaffold region (**C**) 3D reconstruction of the scaffold environment.

To analyse the scaffold volume (SV), a region of interest (ROI) was defined in the middle third of the scaffold height in the form of a cylinder with a diameter of 140 voxels (=4.24 mm) and a height of 60 slices (=1.82 mm), based on established methods [19] (Figure 3A,B).

To examine the scaffold environment, a hollow cylinder (inner diameter: 144 voxels = 4.36 mm; outer diameter: 180 voxels = 5.45 mm; height: 60 slices) was placed around the previously defined ROI (Figure 3A,C). Here, the bone volume (BV), trabecular number (Tb.N), trabecular thickness (Tb.Th) and trabecular spacing (Tb.Sp) were determined. All parameters were evaluated using the µCT Evaluation Program V6.6 software (Scanco Medical, Zurich, Switzerland).

#### 2.4.2. Semiquantitative Evaluation of In Vivo μCT Scans

The semiquantitative evaluation was performed on two levels. To obtain a cross-sectional view of the scaffolds with the surrounding cancellous bone, the original scan was reoriented using the µCT Evaluation Program V6.6 software (Scanco Medical, Zurich, Switzerland). For a more accurate assessment of the degradation process, an established semi-quantitative scoring system was used [19] (Table S1). The following parameters were evaluated by two independent observers in both levels: (1) position in the bone; (2) scaffold–bone contact; (3) distinguishability from bone tissue; (4) degradation behaviour in the medullary and cortical bone; (5) loss of cylindrical shape; (6) formation of a radiolucent reconstruction zone (area within the scaffold volume characterized by a significantly lower gray value). Score values from 0 to 2 were assigned for each parameter.

### 2.5. Micro-Computed Tomography 80 (µCT80)

The distal lateral femoral condyles were separated from the prepared femurs using a diamond band saw (cut-grinder, Walter Messner GmbH, Oststeinbek, Germany) and fixed in 4% formalin solution. They were then dehydrated in an ascending ethanol series (50%, 70%, 80%, 90%, 96% and 100%). The samples were then embedded in methyl methacrylate-based plastic resin (Technovit^®^ 9100, Heraeus Kulzer, Wehrheim, Germany) and, after complete curing, scanned in the even higher resolution µCT80 (Scanco Medical, Zurich, Switzerland) with the settings 70 kV voltage, 600 ms integration time and 10 µm voxel size. The scans were displayed in the same way as the in vivo scans using the µCT Evaluation Program V6.6:403 software (Scanco Medical, Zurich, Switzerland). Manual contouring of the scaffolds and rotation made the cross-section of the original implantation area visible.

#### 2.5.1. Quantitative Analysis of μCT80

For quantitative evaluation, the threshold for cancellous bone (146–272) was determined based on the gray value of eight samples per material group. The BV and bone structure parameters (Tb.N, Tb.Th, Tb.Sp) were analysed in the cylindrical ROI in the middle third of the scaffold area (h = 182 voxels = 1.82 mm; Ø = 424 voxels = 4.24). As a reference for intact cancellous bone, the parameters were determined at the same location in four explanted lateral femoral condyles of adult Zimmermann rabbits with intact femurs.

#### 2.5.2. Semiquantitative Analysis of μCT80

For the semi-quantitative analysis, the entire scaffold area was evaluated by two independent observers in the cross sectional and longitudinal views using a modified scoring system [19]. Scores from 0 to 2 were assigned for the presence of scaffold material and the distribution of trabeculae in the longitudinal and cross sectional view (Table S2).

### 2.6. Histological Examination

From the bone scaffold complexes embedded in Technovit 9100, a central thick section of approx. 70–100 µm was created from the cross-section of the implantation region using the Donath separation thin section technique [31] with a diamond saw (Cut Grinder, Walter Messner, Oststeinbek, Germany) and a grinding machine (Lap Grinder, Walter Messner, Oststeinbek, Germany) and then stained with 0.1% toluidine blue (Waldeck, Münster, Germany). The longitudinal axis of the cylinder was perpendicular to the cut surface.

The histological sections were evaluated by two observers using a light microscope (Zeiss Axio Imager 2, Carl Zeiss Microscopy GmbH, Jena, Germany), the Zeiss Axio Cam Mrc digital camera and the Zeiss ZEN 3.0 software (Carl Zeiss Microscopy GmbH, Jena, Germany) in accordance with the study by Kowalewicz et al. [19].

#### 2.6.1. Quantitative Analysis of the Tissue (Histomorphometry)

Images of the cross sections were taken at 20× magnification from the stained thick sections. A circular ROI (Ø = 1060 pixels = approx. 4.2 mm) was placed centrally around the original implantation area. The percentage areas of scaffold material, newly formed bone, and soft tissue (granulation tissue, bone marrow) were quantified and analysed using the Intellesis module of the Zeiss ZEN 3.0 software(Carl Zeiss Microscopy GmbH, Jena, Germany).

#### 2.6.2. Semiquantitative Analysis of Histological Sections

**Figure 4:**
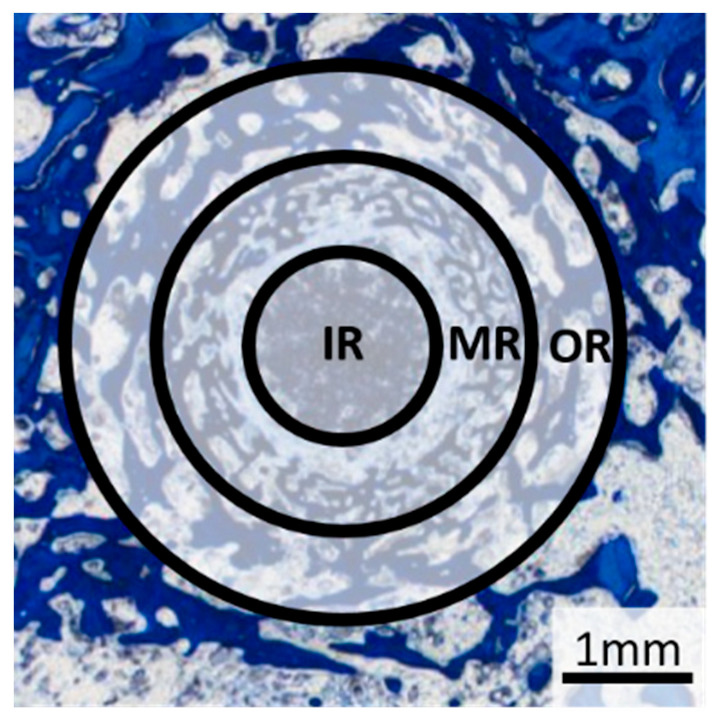
Representation of the subdivision of the defect area for semi-quantitative histological evaluation of the tissue and cells into three zones: IR = inner ring, MR = middle ring, OR = outer ring.

A 25× magnification was used to evaluate the tissue types. Based on the method described by von Doernberg et al. [32] the scaffold region was divided into three zones, each corresponding to one third of the scaffold diameter (inner ring (IR): Ø = 1.41 mm, middle ring (MR): Ø = 2.83 mm, outer ring (OR): Ø = 4.24 mm) (Figure 4). The tissue in each zone was analysed according to the criteria of previous studies [19,32,33].

The scaffold material, young bone tissue (thin, dark blue trabeculae), mature bone tissue (light blue lamellar structure) and the reconstruction zone (cell-rich zone consisting of fibroblasts, macrophages and erythrocyte accumulations) were evaluated according to the area fraction per zone (IR, MR and OR) using scores from 0 to 3: 0% (score 0), 1–25% (score 1), 26–50% (score 2) and over 50% (score 3). Scaffold material surrounded by bone was assessed as follows: no scaffold surrounded by bone (score 0), 1–25% of the scaffold material surrounded (score 1), 26–50% of the scaffold material surrounded, all of the scaffold material surrounded by bone (score 3).

Cell assessment was performed in one field of view per zone at 100× magnification. Adipocytes, precursor cells, macrophages, and osteoblasts were quantified using a semi-quantitative scale (score 0 = none, score 1 = 1–5 cells, score 2 = 6–10 cells, score 3 = >10 cells). Multinucleated cells, including foreign body giant cells (FBGCs) and osteoclast-like cells, were identified based on histological morphology and location (at bone or material surfaces) and evaluated according to the same criteria. Vascular supply was analyzed by the number of erythrocyte accumulations, while osteoid formation was graded as absent (score 0), sporadic (score 1), thin layer on trabeculae/scaffold (score 2), or thick layer on trabeculae/scaffold (score 3).

### 2.7. SEM and EDX Analysis of Scaffold–Bone Composite

For SEM and EDX analysis, a central unstained thin section of the bone-plastic block (4 µm; n = 2 per material and time group) was prepared according to the methods described in Section 2.1.5. The scaffold centre was defined based on the ROIs determined during the histological examination and examined at 28×, 500× and 1000× magnification. The SEM images were used to assess osseointegration and the structure in the implantation area. In the EDX, the presence of material particles was determined based on the distribution of magnesium (Mg), calcium (Ca) and phosphorus (P) ions using EDX.

### 2.8. Statistics

The sample size (n = 8 per material and time point) was determined in accordance with the biometric planning that was approved by the animal experiment authorization, allowing reliable estimation of effect sizes and confidence intervals for exploratory analysis. All statistical evaluations were performed exploratively at a 5% significance level. The statistical analysis of the compressive strength and porosity measurements was performed using 1-way ANOVA analysis and Tukey test (Origin 7G, OriginLab Corporation, Northampton, MA, USA). The in vivo data was evaluated using SPSS Statistics 26 (IBM Company, Armonk, NY, USA). The Shapiro–Wilk test was performed to determine the normal distribution of the quantitative data. Normally distributed data were evaluated using analysis of variance (ANOVA followed by Tukey’s post hoc test). For semi-quantitative and non-normally distributed data, the Kruskal–Wallis test was used, followed by adjustment of the significance values using the Bonferroni correction. Values with *p* ≤ 0.05 were considered statistically significant.

## 3. Results

### 3.1. Scaffold Characterization

#### Physical and Chemical Analysis of Scaffolds

The alkaline post-treatment of the scaffolds with diammonium hydrogen phosphate (DAHP) led to the conversion of farringtonite and magnesium oxide and the formation of struvite. Farringtonite reacted with DAHP and water to form struvite and phosphoric acid (Equation (1)). In addition, magnesium oxide was converted to struvite and ammonia by DAHP and water (Equation (2)).

2 Mg_3_(PO_4_)_2_ + 3 (NH_4_)_2_HPO_4_ + 36 H_2_O → 6 MgNH_4_PO_4_·6 H_2_O + H_3_PO_4_ (1)

MgO + (NH_4_)_2_HPO_4_ + 5 H_2_O → MgNH_4_PO_4_·6 H_2_O + NH_3_ (2)

As already investigated in the preliminary study, stanfieldite shows no chemical reactivity under the given alkaline conditions [18]. The TCP scaffolds consisted almost entirely of ß-TCP, with only small amounts of α-TCP present. The exact percentages of the phases are shown in Table 2.

The compressive strength of the materials investigated varied significantly (*p* ≤ 0.004). Mg3d exhibited the highest compressive strength at 12.5 ± 2.06 MPa, followed by Mg275d at 9.00 ± 1.13 MPa. TCP exhibited the lowest compressive strength at 1.91 ± 0.33 MPa. Young′s moduli for Mg3d, Mg275d, and TCP were calculated to be 1.35 ± 0.58 GPa, 0.69 ± 0.19 GPa, 0.21 ± 0.07 GPa, respectively.

**Figure 5:**
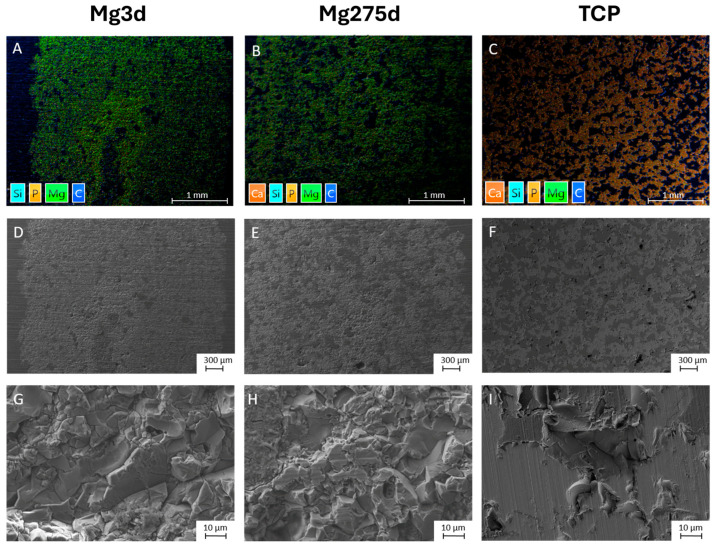
Examination of the scaffolds prior to implantation. EDX images of the scaffolds at 24× magnification (**A**) Mg3d, (**B**) Mg275d and (**C**) TCP. SEM images of the scaffolds at 24× magnification (**D**) Mg3d, (**E**) Mg275d and (**F**) TCP. The pores appear dark gray and the porous scaffold material appears light gray. SEM images at 100× magnification of the scaffolds (**G**) Mg3d, (**H**) Mg275d and (**I**) TCP for assessment of the structural properties.

Mercury intrusion porosimetry revealed an open porosity of 29.2 ± 2.7% for Mg3d, 14.4 ± 2.9% for Mg275d, and 41.8 ± 0.7% for TCP (Table 3). In SEM micrographs, all scaffolds exhibited a porous architecture with distinct morphologies. Mg3d and Mg275d displayed densely packed rhombic crystals forming a rough surface; pores were fine and evenly distributed, with Mg275d showing a slightly higher pore number and local interconnections (Figure 5D,E,G,H). TCP exhibited an amorphous microstructure with larger, partly interconnected pores (Figure 5F,I).

Elemental mapping (EDX) indicated a higher Mg and P signal in the center of Mg3d, whereas Ca, Mg, and P were homogeneously distributed in Mg275d and TCP (Figure 5A–C).

### 3.2. Clinical Examination

In the immediate postoperative period, the rabbits showed a prompt return to normal species-specific behavior and sustained good overall health throughout the study. Two of the 36 animals showed minor lameness lasting up to two days, accompanied by mild wound healing impairment. The disorder, manifesting as erythema and edema in the surgical region, was attributed to wound manipulation by the rabbits and occurred irrespective of the implanted material group. After conservative treatment, the wound healed without complications. All other animals showed physiological wound healing after surgery. There were no clinical abnormalities in the subsequent course of the study.

### 3.3. X-Ray

**Figure 6:**
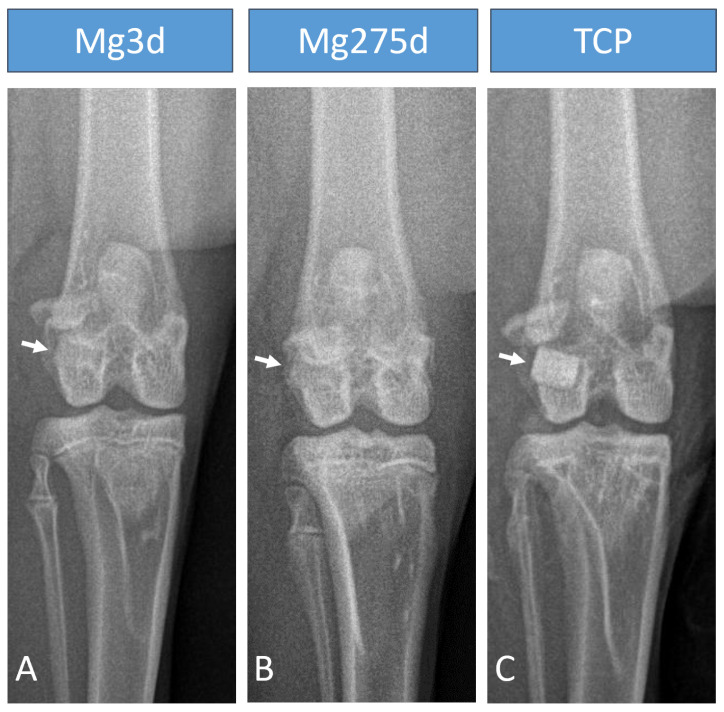
Representative X-ray images (cr/cd view) of rabbit knees immediately after implantation of (**A**) Mg3d, (**B**) Mg275d, and (**C**) TCP. White arrows indicate the implantation sites.

On the day of the operation, X-ray examination revealed a marked difference in the X-ray density of the three materials. Mg275d and Mg3d had a similar X-ray density to the surrounding bone and were only indistinctly distinguishable (Figure 6A,B). TCP was the most clearly distinguishable material from the surrounding bone (Figure 6C). Immediately after surgery, only 17 of 24 Mg3d scaffolds were visible on the cr/cd images and 19 of 24 on the m/l images. At this point, all scaffolds could be detected in both projections for the other two materials. At the following examination times, the visibility of Mg3d and Mg275d decreased rapidly. By week 4, the number of visible Mg3d and Mg275d scaffolds was significantly lower than that of TCP in both projections (*p* < 0.001). By week 16, no Mg3d and Mg275d scaffolds were visible on the cr/cd image. At the same time, two of 16 Mg3d scaffolds and one of 16 Mg275d scaffolds were still visible on the m/l image. TCP scaffolds, on the other hand, were clearly visible until the end of the respective examination period.

### 3.4. In Vivo µCT

#### 3.4.1. Quantitative Analysis of the In Vivo µCT Scan

**Figure 7:**
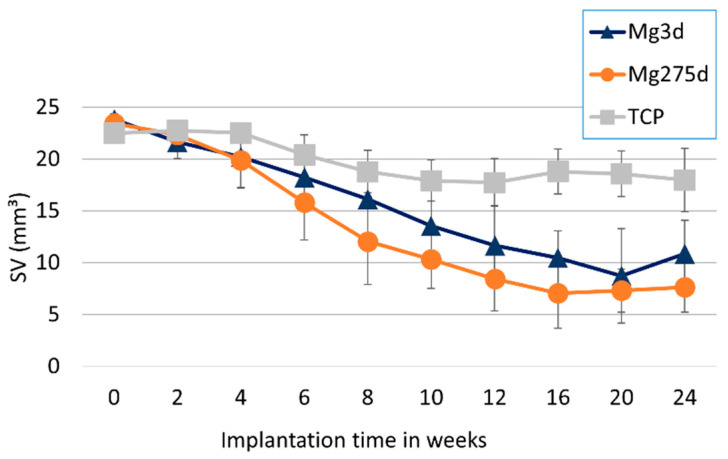
Results of µCT analyses of scaffold volume (SV).

Changes in scaffold volume (SV) of Mg3d, Mg275d, and TCP were monitored over 24 weeks (Figure 7). TCP showed a significantly lower initial SV compared with both MgP-based scaffolds (*p* < 0.001). Mg3d and Mg275d exhibited an early and pronounced reduction during the first weeks, followed by a slower decline thereafter. Between weeks 6 and 12, the degradation rate of Mg275d was significantly higher than that of Mg3d (*p* ≤ 0.02). In contrast, TCP degraded gradually and retained most of its initial volume throughout the study. In week 24, Mg3d had 45.64%, Mg275d 32.57% and TCP 79.86% of the original volume.

**Figure 8:**
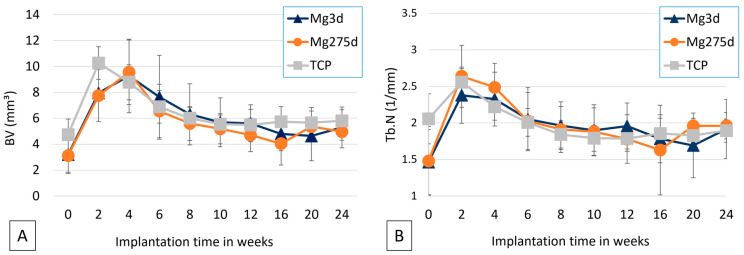
Results of quantitative µCT analysis of (**A**) bone volume and (**B**) trabecular number in the scaffold environment.

Bone volume within the scaffold environment (BV; Figure 8A) increased markedly by week 2 in all groups, with TCP initially showing the highest values (*p* ≤ 0.001). After week 4, inter-group differences disappeared, and BV decreased sharply between weeks 4–8, followed by a slow decline toward late time points.

The trabecular number (Tb.N; Figure 8B) followed a similar temporal pattern across all groups, peaking at week 2. At this time point, Mg275d exhibited a significantly higher Tb.N than Mg3d (*p* = 0.042), whereas no further differences were observed thereafter.

#### 3.4.2. Semiquantitative Evaluation of the In Vivo µCT Scan

All scaffolds were implanted in the spongy area of the lateral femoral condyles with connection to the medullary cavity. By week 2, all implants showed initial bone contact. TCP displayed thin trabecular bridges to the surrounding bone, whereas 9 of 24 Mg3d and 5 of 24 Mg275d scaffolds already exhibited broad contact interfaces. By week 6 at the latest, all materials showed broad contact with the surrounding bone.

Immediately after implantation, all scaffolds were clearly distinguishable from the surrounding bone. However, by week 4, 10 of 24 Mg3d and 7 of 24 Mg275d scaffolds were already showing only indistinct demarcation. This differed significantly from TCP, which remained sharply defined throughout (*p* < 0.001). By week 12, neither Mg3d nor Mg275d scaffolds were distinguishable, while TCP remained visible until the end of the observation period.

The cylindrical contour of Mg3d and Mg275d became blurred from week 4 and was completely lost by week 10, in contrast to TCP, which retained its geometry until study end. This differed significantly from Mg3d (*p* < 0.001) from week 4 onwards and from Mg275d scaffolds (*p* < 0.001) from week 6 onwards.

**Figure 9:**
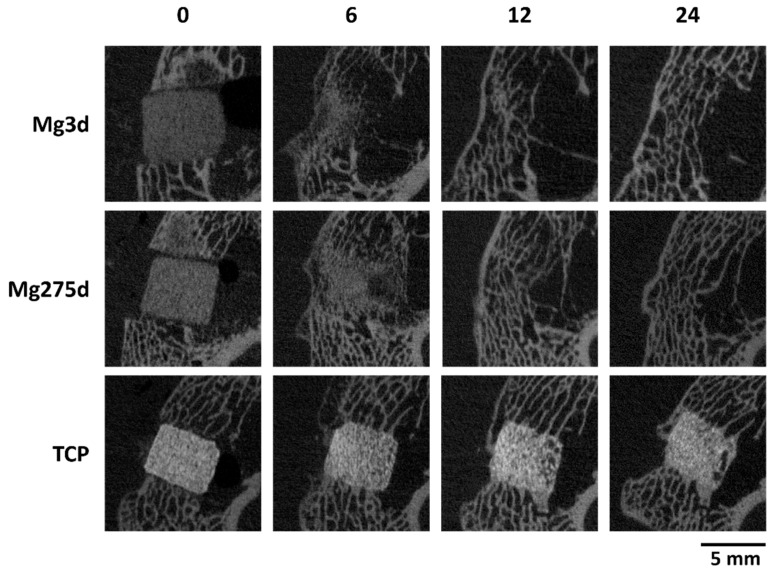
Representative µCT images of Mg3d, Mg275d, and TCP scaffolds at selected time points.

Degradation was consistently more pronounced on the marrow-adjacent side of all scaffolds (Figure 9), particularly in Mg3d and Mg275d. Between weeks 6–10, both materials exhibited significantly more advanced or complete resorption of this portion compared with TCP (*p* ≤ 0.008). Beyond week 12, no residual material could be delineated in µCT. In contrast, TCP showed a slowly increasing degradation of the medullary portion and, in week 12, showed uneven degradation in half of all scaffolds. Complete dissolution of the scaffolds in the medullary area was never observed for TCP.

**Figure 10:**
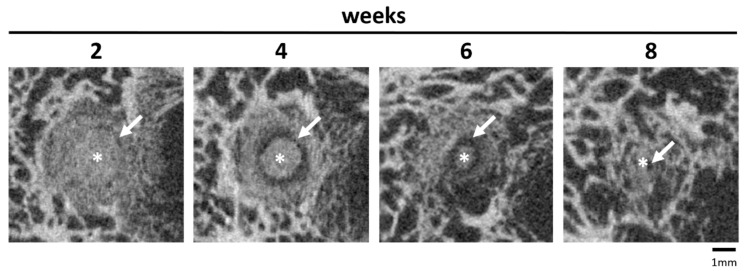
Representative µCT image of the implantation area with a Mg275d scaffold. The asterisk (*) marks the remaining cement core, and the white arrow indicates the radiolucent zone.

In the Mg3d and Mg275d cylinders, a radiolucent zone appeared in the original scaffold area from week 2 onwards (Figure 10). It was most clearly visible in week 4. In the course of the investigations, the zone migrated centripetally towards the centre of the scaffolds and became narrower from week 6 onwards. By weeks 10–12, the zone had completely disappeared, coinciding with the total loss of detectable scaffold structures in the µCT images. A comparable radiolucent zone was only detected in one TCP scaffold in weeks 20 and 24 as an indistinct reconstruction zone.

Overall, Mg3d and Mg275d degraded substantially faster than TCP, with centripetal resorption progressing from the marrow-adjacent regions. Both MgP-based scaffolds showed nearly complete resorption by week 12, whereas TCP retained its geometry throughout the study.

### 3.5. µCT80

#### 3.5.1. Quantitative µCT80 Evaluation

**Figure 11:**
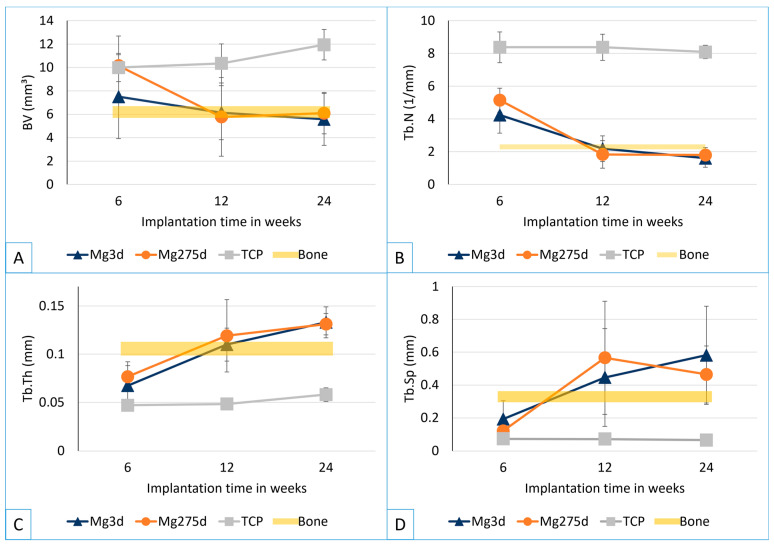
Quantitative micro-computed tomography 80 (µCT80) analyses of (**A**) bone volume (BV), (**B**) trabecular number (Tb.N), (**C**) trabecular thickness (Tb.Th) and (**D**) trabecular separation (Tb.Sp) of the newly formed trabecular bone. The yellow line indicates the area measured in native bone.

Bone volume (BV; Figure 11A) did not differ significantly between groups at week 6. At weeks 12 and 24, TCP showed higher BV than both MgP-based scaffolds (*p* ≤ 0.015), while Mg3d and Mg275d exhibited similar values. For the MgP-based groups, BV declined from week 6 to 24, yet at weeks 12 and 24, both approached the bone volume of the native bone. In contrast, BV measured in TCP continued to increase until study end.

The trabecular number (Tb.N; Figure 11B) was significantly higher for TCP than for the MgP-based scaffolds at all measurement times (*p* ≤ 0.014). For Mg3d and Mg275d, the number decreased significantly between weeks 6 and 12 and then stabilized, reaching levels comparable to native trabecular bone.

The trabecular thickness (Tb.Th; Figure 11C) increased continuously in Mg3d and Mg275d until week 24. In TCP, Tb.Th hardly changed. The trabeculae in Mg275d were significantly thicker than those in TCP from week 6 and in Mg3d from week 12 (*p* ≤ 0.003).

Trabecular spacing (Tb.Sp; Figure 11D) increased over time for the MgP-based scaffolds, whereas TCP remained low and constant (*p* ≤ 0.005).

Despite minor differences in degradation kinetics, Mg3d and Mg275d exhibited comparable bone formation and remodeling behavior, suggesting that moderate variation in Mg content did not markedly influence biological performance.

#### 3.5.2. Semiquantitative µCT80 Evaluation

At week 6, all scaffolds still appeared as connected structures. By week 12, only isolated material fragments were detectable in Mg3d and Mg275d, and by week 24, residual particles were found only in one Mg3d specimen, while Mg275d showed no remaining material. In contrast, TCP scaffolds retained a coherent structure throughout the entire 24-week period.

**Figure 12:**
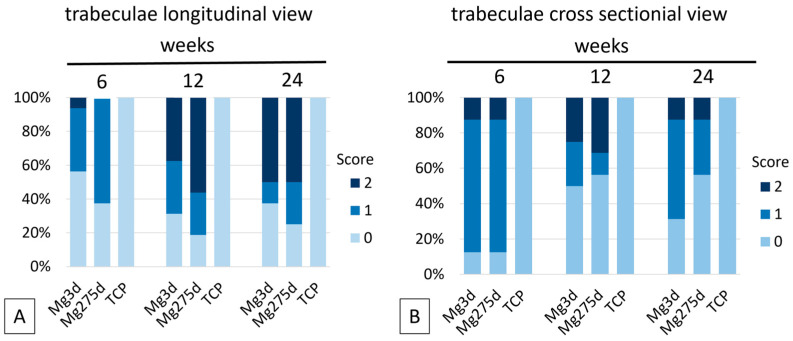
Results of the semi-quantitative µCT80 evaluation of the trabecular structures in the original implantation area in longitudinal view (**A**) and cross sectional view (**B**).

In the longitudinal view, trabecular structures in the marrow-adjacent region of the Mg3d and Mg275d scaffolds became progressively less distinct (Figure 12A). By week 24, only sparse trabecular-like remnants remained in about half of the specimens. This contrasted significantly with TCP, which consistently displayed an even distribution of trabeculae along the entire longitudinal axis at all observation times (*p* ≤ 0.026).

In the cross-sectional view, bone trabeculae had invaded more than half of the scaffold radius in 88% of Mg3d and Mg275d samples by week 6 (Figure 12B). After 24 weeks, complete trabecular bridging of the center occurred in 31% of Mg3d and 56% of Mg275d scaffolds, whereas TCP exhibited uniform trabecular ingrowth across the entire section already from week 6 (*p* ≤ 0.033). These observations indicate slightly faster structural remodeling in Mg275d, although both MgP-based scaffolds showed comparable overall degradation and bone ingrowth patterns.

Notably, histological evaluation confirmed the overall degradation trends observed in µCT, while revealing lower residual scaffold content at late time points, reflecting the similar radiodensity of newly formed bone and scaffold material.

### 3.6. Histology

#### 3.6.1. Histomorphometry

**Figure 13:**
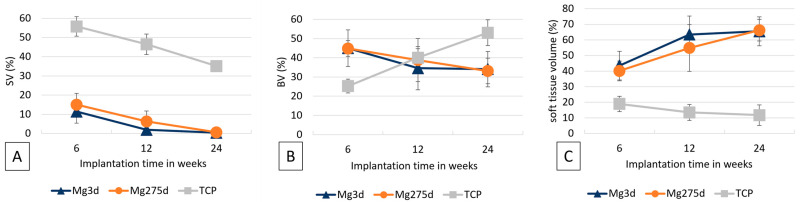
Histomorphometric measurements of thick sections of (**A**) the scaffolds, (**B**) bone and (**C**) soft tissue.

From week 6 onward, the scaffold fraction of Mg3d and Mg275d was significantly smaller than that of TCP (*p* ≤ 0.007, Figure 13A). At this time, the remaining material accounted for only 11.4 ± 6.1% (Mg3d) and 15.0 ± 5.9% (Mg275d) of the measured area, compared with 55.8 ± 5.1% in TCP. After 24 weeks, only traces of material persisted (0.4 ± 0.3% in Mg3d and 0.6 ± 0.4% in Mg275d) whereas TCP still occupied 35.1 ± 1.3% of the area.

The bone content (Figure 13B) increased continuously in TCP, while it declined in the MgP-based scaffolds between weeks 6 and 24. At week 6, TCP showed less bone than the MgP-based groups (*p* < 0.001), but by week 24 it exceeded them (*p* ≤ 0.009) with 53.1 ± 6.7% bone compared with 34.1 ± 9.2% (Mg3d) and 33.2 ± 6.7% (Mg275d).

In contrast to the measured scaffold content, the proportion of soft tissue in the materials behaved differently (Figure 13C). Mg3d and Mg275d contained significantly more soft tissue than TCP at all time points (*p* < 0.001). In the MgP-based groups, this proportion rose steadily to 65.5 ± 9.2% (Mg3d) and 66.2 ± 6.9% (Mg275d) at week 24, whereas TCP showed a slight decrease, reaching only 11.8 ± 6.6% in week 24. These findings confirm the markedly faster resorption of MgP-based scaffolds compared with TCP and their almost complete replacement by soft tissue and immature bone within 24 weeks.

#### 3.6.2. Semiquantitative Analysis of Histology

**Figure 14:**
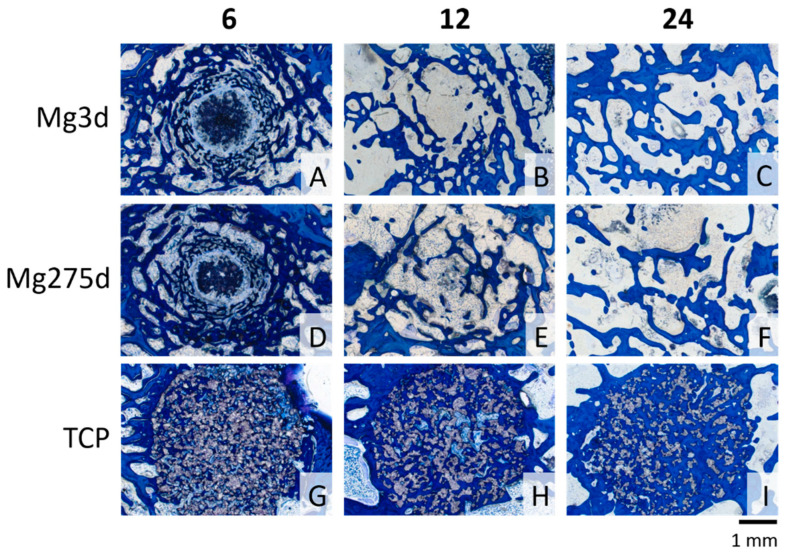
Histological thick sections (toluidine blue staining; magnification ×2.5/0.085) from the center of the scaffolds Mg3d (**A**–**C**), Mg275d (**D**–**F**) and TCP (**G**–**I**) in cross section after 6, 12, and 24 weeks respectively. In toluidine blue staining, the scaffold material remains unstained and appears gray, newly formed bone is visualized in dark blue, and mature/mineralized bone is indicated by a lighter blue shade.

Histological examination of the rings demonstrated distinct degradation patterns between the groups. Mg3d (Figure 14A–C) and Mg275d (Figure 14D–F) exhibited a marked centripetal degradation already by week 6, with scaffold remnants largely restricted to the MR and IR. At week 24, only isolated particles of Mg3d remained in 5 of 8 samples, evenly distributed across all rings, while the other samples were completely devoid of material. Mg275d still exhibited isolated particles in all samples, also homogeneously distributed. In contrast, TCP (Figure 14G–I) showed a uniform distribution of scaffold material across all rings up to week 12, with significantly higher amounts remaining compared to Mg3d and Mg275d (*p* ≤ 0.003). Only at week 24 did TCP show a decline in the OR relative to the MR and IR.

At week 6, bone tissue already surrounded most TCP scaffolds, while in Mg3d and Mg275d, only outer-ring particles were partially encased. Young bone was most abundant in Mg3d, covering more than 50% of the OR area, while Mg275d showed a slightly smaller extent of new bone formation. Toward the IR, bone formation decreased, and in about half of the MgP-based samples, no bone was yet visible. Between weeks 6 and 12, all groups showed a significant increase in well-mineralized mature bone (*p* ≤ 0.019), though only TCP continued to increase thereafter (*p* < 0.001). At week 24, TCP exhibited the highest fraction of mature bone, significantly exceeding Mg275d and Mg3d across all rings (*p* ≤ 0.038). However, small areas of immature bone persisted within the IR in TCP, while Mg3d and Mg275d showed exclusively mature bone.

The amount of soft tissue was significantly greater in Mg3d and Mg275d at weeks 12 and 24 (*p* < 0.001), exceeding 50% of the total area in all rings, whereas TCP contained only 1–25%.

**Figure 15:**
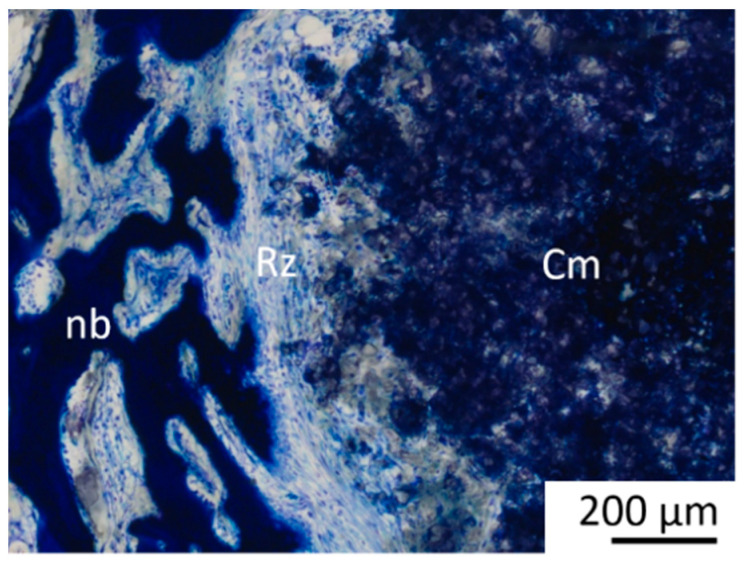
Histological thick section of a Mg3d scaffold in week 6 (magnified ×5/0.15). Newly formed bone (nb)—reconstruction zone (Rz)—remaining scaffold cement (Cm).

A cell-rich radial reconstruction zone consisting of fibroblasts, macrophages and osteoprogenitor cells was present in week 6 in all Mg3d and in 6 of 8 Mg275d scaffolds around the remaining material core in the MR and IR (Figure 15). A similar zone later developed in TCP at week 24. In week 12, no material had a reconstruction zone. This transient, cell-rich reconstruction zone likely represents an active interface where macrophages and osteoprogenitor cells cooperate in scaffold resorption and early bone matrix deposition.

After 6 and 12 weeks, osteoid frequently appeared as a thin rim around the bone trabeculae in all materials. This continued to decrease in Mg3d and Mg275d and was barely present in week 24, while it remained consistently pronounced in TCP.

In week 6, osteoblasts were frequently found in the MR and OR, in accordance with bone formation in Mg3d and Mg275d. In contrast, they were numerous in all rings in TCP.

By week 24, the number of osteoblasts had decreased in all materials, with TCP still showing the highest frequency.

Blood vessels were sparse in Mg3d and Mg275d scaffolds initially but became evenly distributed by week 24, while TCP showed moderate vascularization throughout. Adipocytes increased steadily in Mg3d and Mg275d scaffolds over time, becoming common at week 24, whereas their number in TCP remained the lowest (*p* ≤ 0.009).

Fibroblasts were seen in high numbers in the IR at week 6 in Mg3d and Mg275d. They decreased significantly over time and were no longer detectable at week 24. TCP still had fibroblasts throughout the entire implantation area at week 24, which was significantly different from Mg3d and Mg275d (*p* < 0.001).

The precursor cells were most pronounced in week 12 for all materials. By week 24, their number decreased slightly for all materials. No significant difference was found between the different materials.

Macrophages and FBGCs were most frequent at early time points and decreased markedly in Mg3d and Mg275d, whereas TCP maintained significantly higher levels at all times (*p* ≤ 0.032). Osteoclast-like cells were most abundant in the MR and OR of MgP-based scaffolds initially and declined markedly by week 24, while their distribution in TCP remained constant.

The histological and µCT findings together indicate that bone formation progressed in synchrony with scaffold resorption, with MgP-based scaffolds undergoing rapid remodeling and replacement by newly formed bone, whereas TCP showed slower, continuous mineralization.

### 3.7. EDX- and SEM Examinations

**Figure 16:**
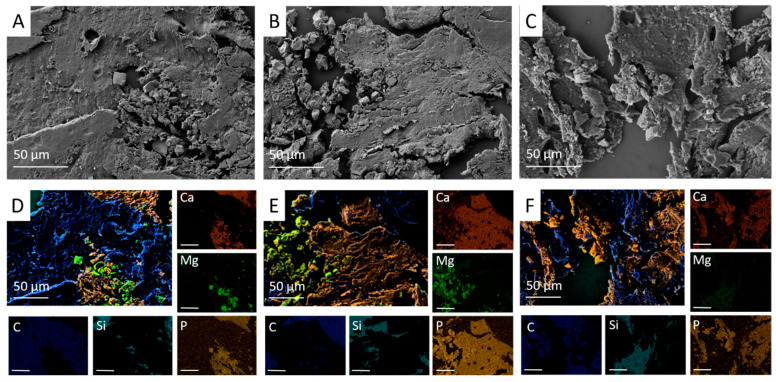
SEM and EDX images of Mg3d (**A**,**D**), Mg275d (**B**,**E**), and TCP (**C**,**F**) at week 6 (magnification ×500). Scaffold residues appear within the implantation area. Scale bar = 50 µm. Elements are color coded as follows: Ca (red), Mg (green), P (yellow), Si (turquoise), and C (dark blue).

EDX and SEM demonstrated a continuous reduction in scaffold material in all groups over time. At week 6, both Mg3d (Figure 16A,D) and Mg275d (Figure 16B,E) still contained notable amounts of residual material in the central region, accompanied by small peripheral particles at the implantation margins. In SEM, these particles appeared smooth-surfaced and seamlessly integrated into the surrounding newly formed bone. In contrast, TCP (Figure 16C,F) displayed distinct, coherent scaffold structures that were clearly separated from host tissue by their higher Ca signal.

By week 12, only a few residual Mg-based particles were detectable, embedded within trabeculae at the periphery of the implantation area. At week 24, merely traces of Mg-containing remnants persisted, located within the bone-marrow spaces. Across all materials, Ca and P were evenly distributed throughout the regenerated bone matrix, while Si signals originated from the glass substrate and C signals from bone-marrow components. These findings confirm the almost complete resorption of MgP-based scaffolds and the homogeneous mineralization of the regenerated bone.

## 4. Discussion

The present study evaluated the degradation, osseointegration, and osteoregenerative capacity of 3D powder-printed magnesium phosphate-based scaffolds (Mg3d and Mg275d) in comparison with TCP as a reference. Both MgP-containing scaffolds exhibited excellent biocompatibility, centripetal degradation that was significantly faster than TCP, and bone formation progressing in synchrony with scaffold resorption. No major differences were found between Mg3d and Mg275d regarding degradation or bone regeneration, indicating that moderate differences in Mg content do not substantially affect biological performance. These findings are consistent with previous CMPC and MPC investigations in rabbits and large animals [17,19,29], confirming the strong osteoconductive response and favorable degradation profile of MgP-based ceramics.

The initial compressive strength of both Mg3d and Mg275d scaffolds exceeded that of TCP and was within the range of cancellous bone [18,34]. This improvement has been attributed to the phase transformation from farringtonite to struvite during DAHP post-treatment [27,35]. Similar strength increases were described for other 3D-printed MPC scaffolds [35,36]. Hemmerlein et al. [29] likewise confirmed adequate stability of 3D-printed CMPC wedges under partial load, emphasizing that the degradation rate must still ensure mechanical integrity until sufficient bone bridging occurs. The measured Young’s moduli in this study (0.21–1.35 GPa) indicate that the tested Ca–Mg–P formulations match the mechanical stiffness of cancellous bone, reducing the risk of stress-shielding. However, since this modulus is substantially lower than cortical bone (≈10–25 GPa), so applications requiring high load-bearing capacity will require metallic reinforcement during the time of bone regeneration. Nevertheless, as no post-implantation mechanical testing was performed, potential changes in mechanical properties during in vivo degradation remain unknown and should be addressed in future studies.

The surface roughness determined for the scaffolds in this study, which is attributed to fused ceramic particles, is a hallmark of 3D powder-printed scaffolds [5,26,37]. This characteristic facilitates a substantial surface area, thereby promoting cell adhesion and early osteointegration [23,38].

In the present study, Mg3d and Mg275d exhibited high open porosity comparable to TCP. Although alkaline post-treatment can slightly reduce porosity via struvite precipitation [27,39], this effect was minimal in the current scaffolds. High porosity supports cell migration and vascularization but compromises mechanical [40]. A comparable porosity range was found optimal for balancing stability and degradation in CMPC and MPC systems [29].

The excellent clinical compatibility observed for the Mg3d, Mg275d, and TCP scaffolds in this study aligns well with prior research on Mg3d-based bone substitutes in various forms [15,41]. Only two rabbits experienced transient mild lameness and minor wound healing issues, which were unequivocally not attributable to the scaffold materials. Moreover, the cellular examination consistently showed no significant inflammatory reaction, further reinforcing the favorable in vivo biocompatibility of the tested materials.

The X-ray examination immediately after surgery showed that Mg3d and Mg275d had similar radiopacity to the surrounding bone, with Mg3d being the least visible. In contrast, TCP could be clearly distinguished from the surrounding bone due to its high X-ray density [42]. Although this limited long-term radiological monitoring, the rapid loss of visibility indicated accelerated degradation. Similar findings have been reported in other in vivo studies, which also observed reduced visibility and faster degradation of CMPC scaffolds compared to TCP [19,41].

µCT analyses confirmed these results, revealing a significantly faster volume loss for Mg3d and Mg275d than TCP from week 4 onwards. Degradation of the MgP-based scaffolds initiated at the periphery and progressed centripetally, leading to an early loss of the cylindrical structure. In contrast, TCP degraded slowly and uniformly, maintaining its geometry until the end of the study. By week 12, Mg3d and Mg275d were barely distinguishable in µCT, although histology and EDX still revealed small remnants, indicating that most degradation occurred within the first 12 weeks. TCP degraded considerably more slowly, consistent with earlier findings [42,43]. Kowalewicz et al. [19] demonstrated almost complete degradation of alkaline-treated MPC scaffolds by week 24, and Kaiser et al. [17] observed similar rapid degradation of struvite-based pastes in vivo.

The accelerated degradation of Mg3d and Mg275d can be attributed mainly to the high solubility of their constituent phases. Stanfieldite (0.361 g/L) and struvite (0.2 g/L) dissolve markedly faster than farringtonite (0.0022 g/L) and the TCP phases α-TCP (0.0025 g/L) and β-TCP (0.0005 g/L) [12,18]. Similar mechanisms were described by Kaiser et al. [17], who reported rapid dissolution of struvite and K-struvite cements in partially loaded sheep tibiae, with up to 63% mass loss after 4 months. As shown in the study by Kanter et al. [10], the highly soluble phases (struvite and stanfieldite) dissolve first, while the loose farringtonite fragments remain. As a result, the scaffolds quickly lose stability [44].

Moreover, degradation in the present study proceeded more rapidly near the bone marrow cavity, where higher vascularization and metabolic activity accelerated ion exchange and dissolution. This region-dependent pattern was more pronounced for Mg3d and Mg275d than for TCP and agrees with prior findings [19,45,46,47].In addition to chemical dissolution, histology showed macrophages, FBGCs and osteoclast-like cells indicating active cell-mediated resorption. Their number decreased as Mg3d and Mg275d degraded, while remaining relatively constant in TCP samples. The appearance of macrophages is considered a typical reaction to cement in rapidly degrading cements [34]. They play an important role in the homeostasis of bone regeneration and are also discussed as osteoclast precursor cells [34]. According to Sheikh et al. [48], the fusion of macrophages into FBGCs is a reaction to the biomaterial. They are formed in the presence of large, difficult-to-degrade particles that cannot be phagocytosed by individual macrophages. Although Gefel et al. [26] reported only chemical dissolution of 3D-printed magnesium phosphate scaffolds composed of struvite and newberyite without osteoclastic resorption in vitro, the present in vivo results demonstrated the presence of osteoclast-like cells at the implantation site, suggesting that cellular mechanisms may contribute to scaffold remodeling under physiological conditions. Such osteoclastic involvement has also been proposed in other in vivo studies [19,28].

These observations support the notion that degradation of MgP-based scaffolds is governed by a synergistic interplay of physicochemical dissolution and cellular resorption. The release of Mg^2+^ and phosphate ions may further promote macrophage activation and osteoclast differentiation. A comparable mixed degradation behavior was also reported for 3D-printed CMPC and MPC wedges in partially loaded rabbit tibiae by Hemmerlein et al. [29], where the presence of FBGCs and osteoclasts coincided with advanced scaffold resorption and early trabecular bone formation.

Minor fluctuations in quantitative µCT data at later time points likely resulted from segmentation artefacts due to similar radiodensity of new bone and residual scaffold. Consequently, µCT may have slightly overestimated residual volume, whereas histology provided more accurate quantification, confirming the overall degradation trend [10,19,49].

A characteristic feature was the formation of a cell-rich reconstruction zone composed of fibroblasts, macrophages, and osteoprogenitor cells. This zone migrated centripetally, drawing a dense network of newly formed trabeculae with it, until it had completely disappeared after 10 (Mg275d) or 12 (Mg3d) weeks at the latest. Similar transient reconstruction zones were described by Kowalewicz et al. [19] for Mg225d scaffolds and by Kaiser et al. [17] for struvite-based MPCs in load-bearing defects. Additionally, cell-rich reconstruction zones of a comparable nature have also been described in rapidly degrading CPCs [42,50]. There is probably a connection between the reconstruction zone and the degradation rate, as it occurs more frequently in faster-degrading cements [17,19]. In addition, the formation of the reconstruction zone may have been favoured by the type of post-treatment. By placing the scaffolds in the DAHP solution, it is possible that the phase transition from farringtonite to struvite occurred most strongly in the peripheral areas of the scaffolds. Due to the higher solubility of struvite compared to farringtonite, the scaffolds degraded more quickly in the peripheral area, making room for migrating cells. The associated high cell activity in the reconstruction zone indicates an active tissue reaction. This zone supports the decomposition of cement and promotes the migration of osteoprogenitor cells and osteoblasts, which contributes to the formation of new bone tissue [17,48].

The faster degradation of Mg3d and Mg275d compared with TCP and the formation of a transient reconstruction zone are promising features, yet their clinical relevance depends on how well degradation and new bone formation are synchronized. The interplay between degradation and bone regeneration is of central importance for the mechanical performance of bioresorbable scaffolds. Too rapid degradation may result in premature loss of stability as fibrous tissue forms around the implant [17,51], whereas excessively slow degradation can delay complete integration and replacement by native bone, potentially promoting foreign-body reactions or chronic inflammation [52].

Histological and µCT80 analyses of the present study revealed material-specific regeneration patterns among the tested materials. In Mg3d and Mg275d scaffolds, bone formation occurred mainly outside the reconstruction zone, forming dense trabecular networks, while TCP exhibited homogeneous bone ingrowth directly onto the scaffold surface, consistent with previous findings by Kowalewicz et al. [19]. All scaffolds showed an early increase in bone volume at week 6 compared with native bone, confirming their osteostimulatory potential, as also documented for other MPC-, CMPC-, and TCP-based materials [15,19,28,41].

The release of Mg^2+^ ions during scaffold resorption likely enhanced osteogenesis and angiogenesis [15,41,53]. Rapid bone formation in the peri-implant region further indicates high biocompatibility [15,16,41,53]. An additional osteoinductive effect may have been triggered by mechanical stimulation during drilling, as described previously [54].

Spatially, MgP-based scaffolds showed greater trabecular formation near the cortex and less in the marrow region, similar to the results reported by Kowalewicz et al. [19] for faster-degrading Mg225d scaffolds. These observations suggest that the rapid degradation of the Mg3d and Mg275d scaffolds in the medullary region could impair their osteoconductive effect. The slower degradation of TCP, on the other hand, preserves sufficient scaffold structure to serve as a guide for bone growth even in the medullary region [19]. However, it should be noted that even in the physiological state, there is less bone tissue in the medullary region, which could further explain the lower trabecular formation in this area.

Between weeks 6 and 24, Mg3d and Mg275d scaffolds exhibited a gradual transition from woven to lamellar bone, with trabecular parameters approaching physiological values. Few osteoblasts or osteoclast-like cells were detected by week 24, indicating completion of bone maturation [19,55]. TCP, in contrast, still contained osteoid and active cells, suggesting delayed remodeling.

Although bone ingrowth was clearly demonstrated, no biomechanical testing of the bone–scaffold composites was performed. Subsequent investigations should therefore include mechanical analyses of explanted specimens to better assess the functional stability and clinical relevance of the regenerated bone. During degradation, macrophages and osteoclast-like cells actively contributed to material resorption, a typical response to bioresorbable magnesium phosphate ceramics [19,34,48].

While this study clearly demonstrates the material-specific osseointegration and degradation behavior of the scaffolds in an unloaded rabbit model, the lack of physiological loading limits direct transferability to clinical conditions. To address this, upcoming experiments should include mechanically more demanding settings and extended observation periods beyond 24 weeks. Evidence from long-term investigations in large animal models demonstrated that complete remodeling and cortical consolidation of magnesium phosphate cements may require up to 9–12 months [10,17,28]. These findings underline the importance of prolonged evaluation to verify the long-term stability and functional performance of regenerated bone in load-bearing applications.

As an exploratory and orientation study, the present work was designed to estimate effect sizes, assess variability, and identify general trends between the different materials. The sample size was determined according to the 3Rs principle, ensuring ethical use of animals while providing meaningful preliminary data. To substantiate the observed tendencies and detect more subtle differences between materials and time points, future studies with larger cohorts will be necessary.

## 5. Conclusions

This study demonstrated that 3D-printed magnesium phosphate-based scaffolds (Mg3d and Mg275d) exhibit rapid, centripetal degradation combined with excellent biocompatibility and early osseointegration in vivo. Both materials showed almost complete resorption within 24 weeks, whereas TCP remained largely intact. The faster degradation of the MgP-containing scaffolds was accompanied by synchronized bone formation and marrow maturation, resulting in the development of mature lamellar bone.

No significant differences were observed between Mg3d and Mg275d in terms of degradation kinetics or osteogenic response, suggesting that the moderate variation in magnesium content between both formulations was insufficient to produce measurable biological differences. A more pronounced alteration in chemical composition may be necessary to achieve distinct degradation and regeneration profiles.

The results highlight the importance of balancing scaffold degradation with new bone formation to maintain mechanical stability during the regeneration process. Although the rapid resorption of Mg3d and Mg275d supports physiological remodeling, excessively fast degradation may risk transient loss of stability before sufficient bone replacement occurs. Histological analyses confirmed the advanced degradation and bone maturation observed radiologically, while also revealing that µCT tended to overestimate residual scaffold material at later stages.

In general, the MgP-based scaffolds exhibited superior biological performance in comparison with TCP, thus indicating their potential as promising candidates for resorbable bone substitute applications. It is recommended that future research encompass biomechanical testing and evaluation under load-bearing conditions, with the objective of validating their functional performance in clinically relevant scenarios.

## Figures and Tables

**Table 1:** Molar chemical composition of cement raw powders.

Ca_x_Mg_3−x_(PO_4_)_2_	CaHPO_4_	CaCO_3_	MgHPO_4_·3H_2_O	Mg(OH)_2_
x = 0.00	-	-	2.00	1.00
x = 0.25	0.17	0.08	1.83	0.92
x = 3.00	2.00	1.00	-	-

**Table 2:** Chemical composition of the scaffolds after sintering and after treatment with DAHP in wt%.

	Farringtonite	Struvite	Stanfieldite	Periclase	α-TCP	ß-TCP
Mg3d	79.9 ± 2.7	20.1 ± 2.7	-	-	-	-
Mg275d	66.2 ± 2.2	11.7 ± 0.1	17.2 ± 1.0	4.9 ± 3.0	-	-
TCP	-				1.5 ± 0.8	98.5 ± 0.8

**Table 3:** Porosity measured using mercury porosimetry in %.

	Open Porosity	Closed Porosity	Total Porosity
Mg3d	29.2 ± 2.7	15.4 ± 5.2	44.7 ± 2.5
Mg275d	14.4 ± 2.9	22.4 ± 4.1	36.8 ± 5.7
TCP	41.8 ± 0.7	1.7 ± 0.9	43.5 ± 1.6

## Data Availability

The original contributions presented in the study are included in the article, further inquiries can be directed to the corresponding author.

## Supplementary Materials

The following supporting information can be downloaded at: https://www.mdpi.com/article/10.3390/ma18225067/s1, Table S1: Scoring system for semiquantitative µCT analysis; Table S2: Scoring system for semiquantitative µCT80 trabecular analysis.

## Abbreviations

The following abbreviations are used in this manuscript:

µCT	microcomputed tomography
µCT80	micro-computed tomography 80
BV	bone volume
CMPCs	calcium magnesium phosphate cements
CP	calcium phosphate
CPCs	calcium phosphate cements
cr/cd	cranio-caudal
FBGCs	foreign body giant cells
IR	inner ring
m/l	medio-lateral
MgP	magnesium phosphate
MPCs	magnesium phosphate cements
MR	middle ring
OR	outer ring
ROI	region of interest
SV	scaffold volume
Tb.N	trabecular number
Tb.Sp	trabecular spacing
Tb.Th	trabecular thickness
Th	threshold
