# Supplementary material for: In Vivo Degradation Behaviour and Osteoregenerative Capacity of 3D-Printed Magnesium Phosphate and Calcium Magnesium Phosphate Cement Scaffolds
Source: Materials (Basel). 2025 Nov 7;18(22):5067. doi: 10.3390/ma18225067 (PMC12654012; doi:10.3390/ma18225067)
Supplement: Supplementary file 1 [file materials-18-05067-s001.zip › materials-3915624-supplementary.pdf]

materials

MDPI

Supplementary Materials for:

In Vivo Degradation Behaviour and Osteoregenerative Capacity of 3D-Printed Magnesium Phosphate and Calcium Magnesium Phosphate Cement Scaffolds

Sophia Hiepe [1,*], Elke Vorndran [2], Franziska Feichtner [1], Anja-Christina Waselau [1] and Andrea Meyer-Lindenberg [1]

1 Clinic for Small Animal Surgery and Reproduction, Ludwig Maximilians University Munich, 80539 Munich, Germany; a.waselau@lmu.de (A.-C.W.); ameylin@lmu.de (A.M.-L.)
2 Department for Functional Materials in Medicine and Dentistry, University of Wuerzburg, 97070 Wuerzburg, Germany; elke.vorndran@fmz.uni-wuerzburg.de (E.V.)
* Correspondence: s.hiepe@lmu.de;

Supplementary Table S1. Scoring system for semiquantitative µCT analysis.

Parameters	Score 0	Score 1	Score 2
Scaffold-bone-contact	Broad contact area between scaffold and bone, numerous bone trabeculae on scaffold/ no gap visible	Multiple bone trabeculae between scaffold and surrounding bone/ barely visible gap	No contact between scaffold and surrounding bone/ clear gap between bone and scaffold
Scaffold demarcation	Scaffold cannot be distinguished from the surrounding bone tissue	Scaffold partially distinguishable from surrounding bone tissue	Scaffold completely distinguishable from surrounding bone tissue
Degradation behavior	Scaffold uniformly degraded	Degradation was more pronounced in the scaffold region adjacent to the bone marrow	Complete Degradation of the scaffold region adjacent to the bone marrow
Loss of shape	Cylindrical shape no longer recognizable	Cylindrical shape partially recognizable	Cylindrical shape clearly recognizable
Reconstruction zone (area of significantly lower threshold within the scaffold volume)	No reconstruction zone	Reconstruction zone indistinctly delineated	Reconstruction zone distinctly delineated

materials

MDPI

Supplementary Table S2. Scoring system for semiquantitative μCT80 trabecular analysis.

Parameters	Score 0	Score 1	Score 2
longitudinal view	Trabecular structures throughout the entire scaffold volume	More trabeculae near the cortex than the medulla	Trabeculae predominantly near cortex; few near medulla
cross-sectional view	Numerous trabecular-like structures visible up to the center of the scaffold radius	Trabecular structures visible >50% of scaffold radius, not reaching the center	Trabecular structures visible in <50% of the scaffold radius
